# Downregulated USP3 mRNA functions as a competitive endogenous RNA of SMAD4 by sponging miR-224 and promotes metastasis in colorectal cancer

**DOI:** 10.1038/s41598-017-04368-3

**Published:** 2017-06-27

**Authors:** Zaozao Wang, Jie Yang, Jiabo Di, Ming Cui, Jiadi Xing, Fan Wu, Wei Wu, Hong Yang, Chenghai Zhang, Zhendan Yao, Nan Zhang, Beihai Jiang, Xiangqian Su

**Affiliations:** Key Laboratory of Carcinogenesis and Translational Research (Ministry of Education), Department Gastrointestinal Surgery IV, Peking University Cancer Hospital & Institute, Beijing, 100142 China

## Abstract

Increasing evidence shows that competitive endogenous RNAs (ceRNAs) can affect the expression of other transcripts by sequestering common microRNAs (miRNAs), and participate in tumourigenesis. As a potent tumour suppressor in colorectal cancer (CRC), SMAD4 is regulated by many miRNAs. However, the regulation of SMAD4 by ceRNAs has never been examined. In the present study, we found that USP3 modulated SMAD4 expression in a miRNA dependent, and protein-coding gene independent manner. USP3 and SMAD4 were directly targeted by miR-224, and overexpression of the USP3 3′UTR could inhibit metastasis caused by the loss of USP3. The correlation of USP3, SMAD4 and miR-224 expression was further verified in CRC specimens. Additionally, the loss of USP3 was associated with distal metastasis and a poor prognosis. Altogether, our study demonstrates USP3 as a bona fide SMAD4 ceRNA. The results from this study may provide new insights into the prevention and treatment of CRC.

## Introduction

CRC is the fourth leading cause of cancer mortality worldwide^1^, representing a major public health problem. Metastasis remains the ultimate cause of cancer-related death in most CRC cases^2^, and accounts for a 5-year survival rate of less than 10% for CRC patients with distal dissemination^3^. Hence, it is vital to uncover the critical molecular events involved in CRC metastasis.

As a potent tumour suppressor gene, SMAD4 inhibits CRC metastasis and is often found to be deleted in the late stage of adenoma-carcinoma sequence in colorectal carcinogenesis^3, 4^. Loss of SMAD4 facilitates CRC metastasis through different signalling pathways^5–7^ and correlates with a significantly reduced survival and a poor response to chemotherapy in the clinic^8–11^. Moreover, nearly 54% LOH (18q21) and a frequency of 8.6% for various types of mutation could be detected at the SMAD4 locus in sporadic CRC^12^. Recent studies have demonstrated that miRNAs also contribute to SMAD4 inactivation^13–17^.

miRNAs are small non-coding RNAs which regulate gene expression through the degradation of transcripts or by interfering with protein translation. They form complicated post-transcriptional networks with multiple RNA transcripts^18–20^. Based on these networks, a new layer of miRNA dependent gene regulation has recently been defined as ceRNA crosstalk. ceRNAs refer to specific RNAs harbouring the same set of miRNA response elements (MREs) which regulate and communicate with each other by competing for a limited pool of miRNAs^21, 22^. An increasing number of studies have demonstrated that ceRNA crosstalk is essential for gene regulation^23, 24^, and perturbations of ceRNA networks play important roles in tumour biology^25–28^. Through sequestrating miRNAs, ceRNA molecules may emerge as novel therapeutic tools in cancer treatment^29^. During CRC development, ectopically expressed ceRNAs participate in tumour formation through titrating miRNAs away from the 3′ untranslated regions (3′UTR) of protein-coding genes^27, 28, 30–32^.

As a central signalling transducer of the TGF-β pathway, SMAD4 has been validated as a direct target of several oncogenic miRNAs, including miR-224^13–15^, miR-20a-5p^16^ and miR-130a/301a/454^17^ in CRC. However, it is unclear whether specific ceRNAs exist that can modulate SMAD4 expression by competing for shared miRNAs to participate in CRC progression.

In the present study, ubiquitin specific peptidase 3 (USP3), a member of mammalian deubiquitinating enzymes (DUBs), was identified as a ceRNA of SMAD4 by computational analysis. After confirming the crosstalk between USP3 and SMAD4, subsequent experiments verified that the USP3 3′UTR could regulate SMAD4 expression by sponging miR-224, which resulted in the inhibition of CRC cell metastasis. Furthermore, USP3 expression was decreased in CRC tissues. Loss of USP3 not only facilitated distal metastasis but also indicated a poor prognosis in CRC patients. To our knowledge, this is the first study to demonstrate that the USP3 3′UTR acts as a SMAD4 ceRNA through competing for miR-224, and the regulatory mechanism may provide novel perspectives on the diagnosis and treatments of patients with CRC.

## Results

### Identification of SMAD4 ceRNA candidates

To identify SMAD4 ceRNAs in CRC, the approach termed mutually targeted microRNA response element enrichment (MuTaME) was used^33, 34^. A series of bioinformatics analyses combined with experimental screening and Gene Expression Omnibus (GEO) datasets validation were performed.

Firstly, it has been well established that there are 14 validated SMAD4-binding miRNAs, including miR-224^13–15^ (Supplementary Fig. S1a). According to MuTaME and the stringent criteria^33, 34^, the rna22 microRNA target prediction algorithm and Starbase V2.0 were performed to predict the microRNA target protein-coding transcriptome, also known as candidate SMAD4 ceRNAs. The 3′UTR of a candidate ceRNA should be targeted by at least 8 microRNAs^33, 34^. To further narrow down candidate ceRNAs, the 3′UTR of the predicted ceRNAs should also contain the MREs of 5 validated SMAD4-targeting miRNAs derived from CRC, including miR-224^13–15^, miR-20a-5p^16^, miR-130a-3p^17^, miR-301a-3p^17^ and miR-454-3p^17^. Thus, 82 candidate ceRNAs were available through the preliminary prediction (Supplementary Fig. S1a).

Next, a PubMed literature review and an Oncomine database analysis were used to identify candidate ceRNAs that were differentially expressed in CRC. 14 out of 82 putative SMAD4 ceRNAs were obtained. We then determined the ability of SMAD4 to modulate the levels of these candidate SMAD4 ceRNAs by evaluating the effects of endogenous SMAD4 depletion on the levels of 14 candidates in CRC cells. qRT-PCR analyses suggested that knockdown of SMAD4 could induce significant decreases in the levels of USP3, QKI, and PAFAH1B1 transcripts. The results raised the possibility that these 3 targets were SMAD4 ceRNAs (Supplementary Fig. S1b).

To confirm whether USP3, QKI and PAFAH1B1 were SMAD4 ceRNAs, four CRC datasets were applied to analyse the correlation between SMAD4 and these candidates. The significant correlation between USP3 expression and SMAD4 expression was detected in all of the four CRC datasets (Supplementary Fig. S1c). However, the expression of QKI and PAFAH1B1 were only correlated with the SMAD4 transcript in partial CRC datasets (Supplementary Fig. S1d,e). Besides, USP3 was considered to have no direct protein-protein interactions with SMAD4 by the mentha database^35^. Therefore, USP3 was chosen as a SMAD4 ceRNA for our further study.

### USP3 modulated SMAD4 expression at the post-transcriptional level

**Figure 1:**
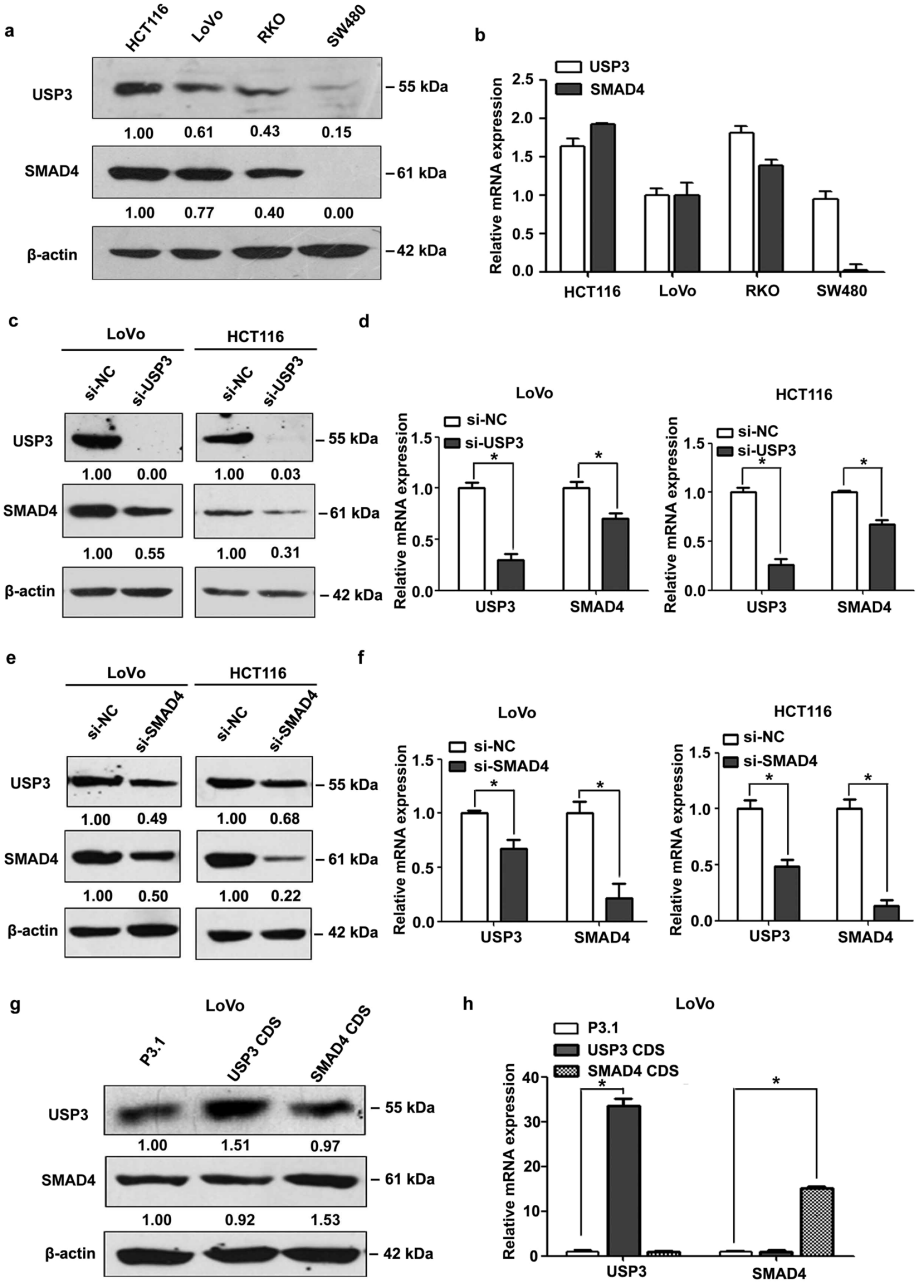
USP3 interacted with SMAD4 at the post-transcriptional level. (**a,b**) Protein and mRNA expression of USP3 and SMAD4 were examined by western blot and qRT-PCR in CRC cells. (**c,e**) USP3 and SMAD4 protein and (**d,f**) mRNA levels were decreased after knockdown of either USP3 or SMAD4 by transfection of siRNA pools in LoVo and HCT116 cells, respectively. (**g,h**) Overexpression of the USP3 or SMAD4 CDS did not affect the other’s expression at both protein and mRNA levels. All values are represented as the mean ± SD (n = 3). Western blot images are cropped; full-length blots are included in Supplementary Figures S7 to S10. The densitometric measurement was performed with each western blot analysis; relative protein expression was normalized to β-actin. *P < 0.05.

To investigate the correlation between USP3 and SMAD4, the protein and mRNA expression of these two molecules were examined by western blot and qRT-PCR in CRC cells. The expression patterns of USP3 and SMAD4 were similar at both the protein and the mRNA levels. SMAD4 expression was undetectable in SW480 cells as previously reported^36^ (Fig. 1a,b). To further ascertain the interdependence of USP3 and SMAD4, siRNA-mediated gene silencing was performed in CRC cells with an siRNA pool (Supplementary Fig. S2). Silencing of USP3 resulted in decreased SMAD4 protein expression in LoVo and HCT116 cells (Fig. 1c), so did SMAD4 mRNA (Fig. 1d). Similarly, knockdown of SMAD4 attenuated USP3 expression at both the protein (Fig. 1e) and the mRNA (Fig. 1f) levels. In addition, expression changes of the USP3 and SMAD4 mRNA in the gene knockdown experiments indicated that the more effective the silencing of one transcript, the more powerful the expression reduction of the other. What’s more, overexpression of the USP3 coding sequence had little effect on SMAD4 expression at either the mRNA or the protein levels, or vice versa (Fig. 1g,h). Briefly, these results indicated that SMAD4 and USP3 crosstalk can affect the expression of each other at the post-transcriptional levels.

### Regulation of SMAD4 by USP3 was 3′UTR and miRNA dependent

**Figure 2:**
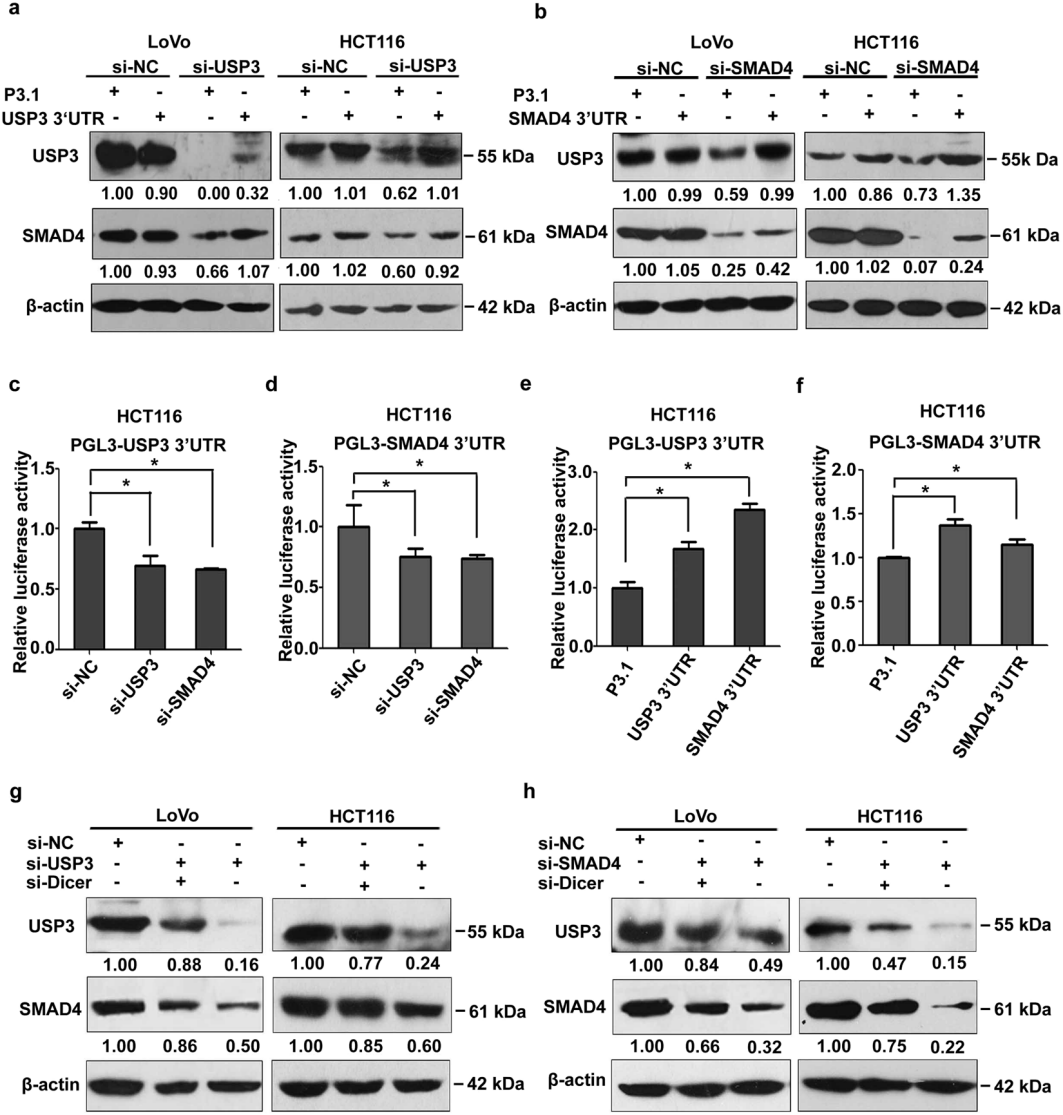
Crosstalk between USP3 and SMAD4 was 3′UTR and miRNA dependent. (**a,b**) USP3 3′UTR or SMAD4 3′UTR mutually modulated protein expression of the other when endogenous USP3 or SMAD4 expression was first reduced by siRNA transfection. (**c,d**) Deletion of USP3 or SMAD4 attenuated luciferase activity of the PGL3-USP3 3′UTR or PGL3-SMAD4 3′UTR reporters; (**e,f**) overexpression of USP3 3′UTR or SMAD4 3′UTR increased the luciferase activity of the corresponding reporter plasmids. (**g,h**) USP3 and SMAD4 expression was elevated when Dicer depletion was combined with USP3 or SMAD4 knockdown, compared with treatment of si-USP3 or si-SMAD4 alone. All values are represented as the mean ± SD (n = 3). Western blot images are cropped; full-length blots are included in Supplementary Figure S11 to S14. Quantitative protein expression data were normalized to β-actin. *P < 0.05.

To validate that USP3 acted as a ceRNA of SMAD4, USP3 and SMAD4 3′UTR expressing plasmids were constructed. Although protein expression of SMAD4 and USP3 could not be elevated by the transfection of their corresponding 3′UTRs, they could be upregulated when the endogenous expression of USP3 or SMAD4 was initially knocked down in CRC cells (Fig. 2a,b).

ceRNAs and transcripts of protein-coding genes were reported to regulate each other by sponging the same miRNAs that target both 3′UTRs. We utilized luciferase reporter constructs of PGL3-USP3 3′UTR and PGL3-SMAD4 3′UTR to evaluate whether the interaction between USP3 and SMAD4 was 3′UTR dependent. PGL3-USP3 3′UTR or PGL3-SMAD4 3′UTR was ectopically expressed in HCT116 cells, and USP3 or SMAD4 was subsequently knocked down. Silencing either USP3 or SMAD4 resulted in diminished PGL3-USP3 3′UTR or PGL3-SMAD4 3′UTR activity (Fig. 2c,d), which raised the possibility that the availability of common miRNAs was elevated when either USP3 or SMAD4 was suppressed. Similarly, overexpression of USP3 or SMAD4 3′UTR led to significantly increased activities on the PGL3-USP3 3′UTR and PGL3-SMAD4 3′UTR reporter constructs (Fig. 2e,f). The effects were probably due to competition for common miRNAs.

Based on the essential function of Dicer in the miRNA biogenesis pathway^37^, we silenced Dicer using siRNAs to further confirm whether the interdependence between USP3 and SMAD4 was mediated by miRNAs^38, 39^. The knockdown efficiency of Dicer and its effect on miRNA expression were confirmed by qRT-PCR (Supplementary Fig. S3). As expected, the expressions of USP3 and SMAD4 were both upregulated when Dicer was knockdown in the CRC cells with depletion of USP3 or SMAD4 (Fig. 2g,h), indicating that crosstalk between USP3 and SMAD4 was abolished after Dicer was silenced. These results demonstrated that the regulation of SMAD4 by USP3 was dependent on their 3′UTRs and, specifically, on their shared miRNAs.

### USP3 and SMAD4 were direct targets of miR-224

To elucidate which miRNA targeted both SMAD4 and USP3 to mediate their crosstalk, microRNA.org and TargetScan were used to search for common MREs between their 3′UTRs. We identified that the SMAD4 and USP3 3′UTRs shared 22 common miRNA binding sites, and contained 24 and 23 MREs for these miRNAs, respectively. Among them, miR-224 exhibited the highest mirSVR scores for both SMAD4 and USP3, suggesting its greatest potential for stable binding and efficient suppression of target genes (Supplementary Table S2). Moreover, the MREs that were located in 1047–1053 bp of SMAD4 3′UTR and 134–140 bp of USP3 3′UTR were predicted to be the most potent response elements for miR-224 according to the mirSVR scores (Supplementary Table S3).

**Figure 3:**
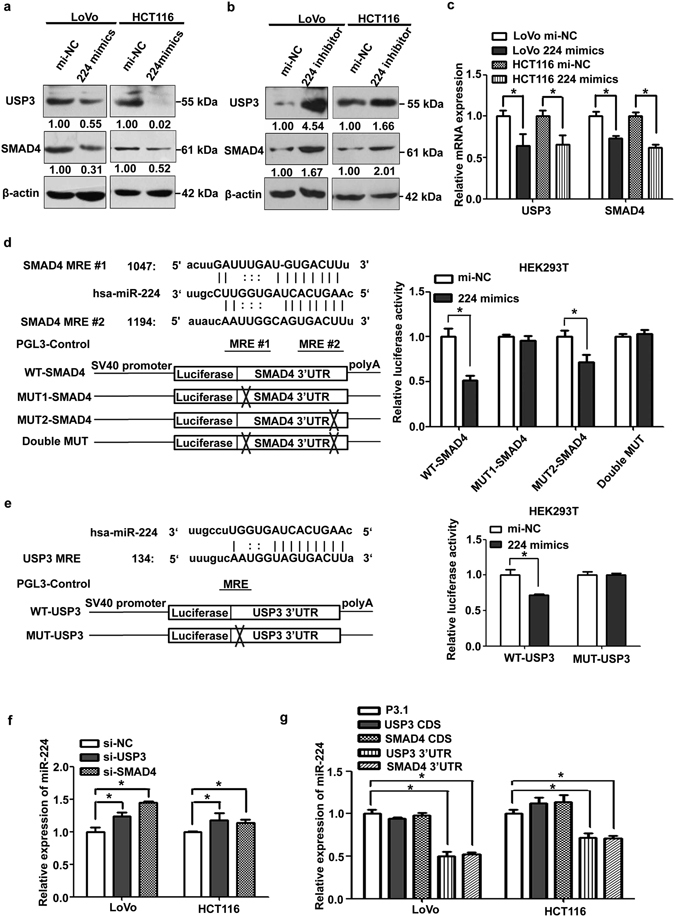
miR-224 modulated expression of USP3 and SMAD4. (**a**) miR-224 mimics attenuated protein expression of USP3 and SMAD4 and (**b**) miR-224 inhibitor enhanced USP3 and SMAD4 expression. Western blots are cropped; full-length blots are included in Supplementary Figures S15 and S16. The results were quantified by ImageJ software, and the relative protein ratio was normalized to β-actin. (**c**) Expression of USP3 and SMAD4 mRNA were reduced by miR-224 mimics in LoVo and HCT116 cells. (**d,e**) Left upper panel: Schematic diagram of miR-224-binding sites in the SMAD4 or the USP3 3′UTRs from microRNA.org. Left lower panel: Construction of USP3 or SMAD4 luciferase reporter plasmids containing wild-type miR-224 MREs (WT) or mutant sequences (MUT). Right panel: Luciferase reporters harbouring wild-type or mutant miR-224-binding sites of the USP3/SMAD4 3′UTR were co-transfected with miR-224 mimics/mi-NC. Relative luciferase activity is presented as the mean ± SD (n = 3). (**f,g**) miR-224 expression was determined by qRT-PCR after USP3/SMAD4 silencing or USP3/SMAD4 CDS or 3′UTR overexpression in CRC cells. *P < 0.05.

miR-224 has been validated as an oncogenic miRNA which promotes CRC metastasis by targeting SMAD4^13–15^, but its regulation of USP3 has not yet been characterized. To verify the post-transcriptional regulatory effects of miR-224 on USP3 and SMAD4, miR-224 mimics or inhibitors were used. The endogenous expression level of miR-224 and the transfection efficiency of miR-224 mimics or inhibitors were confirmed in CRC cells (Supplementary Fig. S4b). We found the protein levels of both USP3 and SMAD4 were decreased after miR-224 was overexpressed (Fig. 3a) and increased when miR-224 expression was reduced (Fig. 3b), compared with cells transfected with mi-NC. In addition, USP3 and SMAD4 mRNA expression were significantly suppressed by miR-224 mimics in CRC cells (Fig. 3c).

To determine whether SMAD4 and USP3 were bona fide targets of miR-224, 3′UTR fragments of SMAD4 and USP3 containing wild-type or mutant miR-224 binding sites were introduced downstream of the luciferase coding region of the PGL3-control reporter construct (Fig. 3d). HEK293T cells were then co-transfected with corresponding reporter plasmids and miR-224 mimics/mi-NC. After the treatments, the luciferase activity of the PGL3-WT SMAD4 3′UTR construct was significantly suppressed (Fig. 3d) compared with the unaffected activity of the reporter plasmid containing mutant miR-224 binding site 1 in SMAD4 3′UTR (MUT1-SMAD4). The construct containing mutant miR-224 binding site 2 in SMAD4 3′UTR (MUT2-SMAD4) showed moderately reduced luciferase activity (Fig. 3d). Additionally, the reporter construct with the double mutant binding sites of SMAD4 3′UTR was not affected by the transfection of the miR-224 mimics (Fig. 3d). Furthermore, co-transfection of the miR-224 mimics with the reporter plasmid reduced the luciferase activity of the PGL3-WT USP3 3′UTR construct but not the mutant that harboured the sequence without the miR-224 response elements (Fig. 3e). Therefore, SMAD4 and USP3 were direct targets of miR-224, and the first MRE of SMAD4 was the key interaction site for miR-224, which was consistent with the previous study by Ling *et al*.^13^.

Furthermore, to validate that the ceRNA crosstalk between USP3 and SMAD4 was mediated by miR-224, miR-224 expression was evaluated after either of the two transcripts was silenced or when the corresponding 3′UTR/CDS was overexpressed. miR-224 was clearly increased after the knockdown of either USP3 or SMAD4 in LoVo and HCT116 cells (Fig. 3f). While, the ectopically expressed SMAD4/USP3 3′UTR significantly decreased miR-224 expression in both cell lines. However, the level of miR-224 was unchanged by the transfection of USP3/SMAD4 CDS (Fig. 3g).

In summary, these results indicated that USP3 and SMAD4 formed a symmetrical ceRNA network through miR-224 in CRC cells.

### USP3 knockdown-induced metastasis was abolished by USP3 3′UTR overexpression in CRC cells

**Figure 4:**
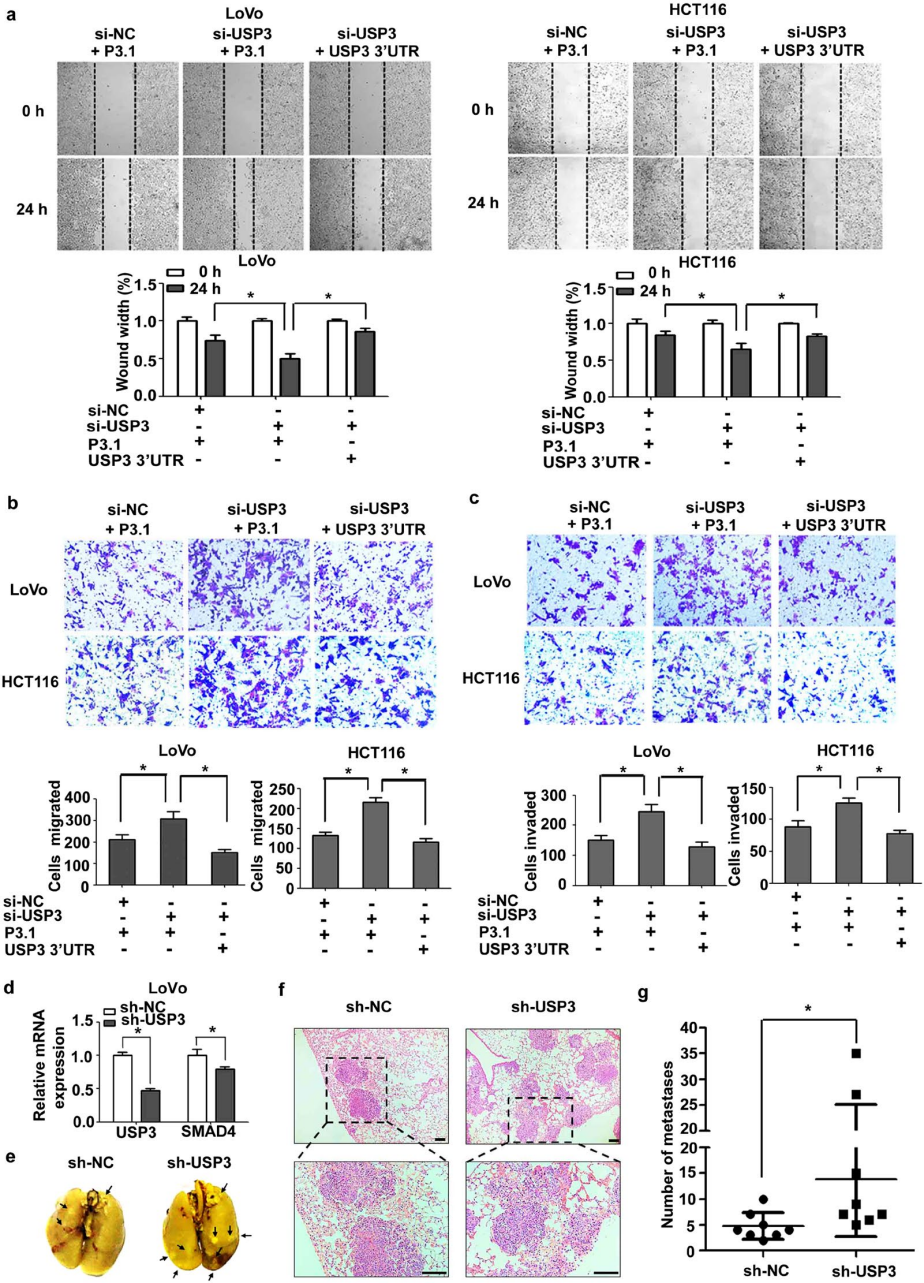
Reduced USP3 expression promoted migration and invasion of CRC cells *in vitro* and *in vivo*. (**a**) Wound-healing assays were performed to detect CRC cell migration after transfection with si-USP3 or co-transfection with si-USP3 and USP3 3′UTR. Photographs were obtained 0 and 24 h after scratching. (**b,c**) Transwell assays with or without Matrigel coatings were used to evaluate migration and invasion by LoVo and HCT116 cells after the indicated transfection treatment. The migratory or invasive cells were stained with crystal violet, and counted under a microscope in five random fields at 100 × magnification. Quantification data are shown as the mean ± SD (n = 3). *P < 0.05. (**d**) After transduction of the sh-USP3 lentivirus, mRNA expression of USP3 and SMAD4 were both reduced in LoVo cells compared with the group transduced with sh-NC lentivirus. (**e**) Representative photographs of lungs stained with picric acid, which were harvested from NOD/SCID mice eight weeks after injection with stably transduced sh-NC or sh-USP3 LoVo cells (2 × 10^6^ cells per injection) via tail veins (n = 8); arrows indicate metastatic tumours. (**f**) Representative images of haematoxylin and eosin-stained sections in sh-NC and sh-USP3 lung metastatic foci. Scale bars, 100 μm. (**g**) The number of lung metastatic nodules was counted under a microscope. The results are presented as the mean ± SD (n = 8), *P < 0.05.

To further investigate the effects of USP3 and its 3′UTR on metastasis, wound-healing and transwell assays were conducted to examine the migration and invasion capabilities of CRC cells. USP3 knockdown promoted higher motility of LoVo and HCT116 cells in the wound-healing process after scratching (Fig. 4a). Moreover, depletion of USP3 resulted in an elevated invasive activity in CRC cells (Fig. 4b,c). However, overexpression of the USP3 3′UTR abrogated the promoting effect of USP3 silencing on cell migration and invasion (Fig. 4a–c). These results indicated that the loss of USP3 facilitated tumour progression, while overexpression of the USP3 3′UTR inhibited metastasis in CRC cells.

In addition, an *in vivo* metastasis experiment was performed. LoVo cells transduced with the sh-USP3 or the control lentivirus were injected intravenously into NOD/SCID mice through the tail vein. The transduction efficiency was evaluated by qRT-PCR, and the results showed that SMAD4 mRNA was decreased when USP3 was stably knockdown (Fig. 4d). Animals were sacrificed 8 weeks after injection. The lungs of mice in the sh-USP3 group had more metastatic colonies either by gross observation (Fig. 4e) or under a light microscope (Fig. 4f) than those of the sh-NC group. Micrometastases in each lung were counted, and the number of metastatic foci in the lung tissues was significantly increased in the sh-USP3 group compared with the control group (Fig. 4g), indicating the anti-metastatic capability of USP3.

### Functional crosstalk of USP3, SMAD4 and miR-224 in human CRC specimens

**Figure 5:**
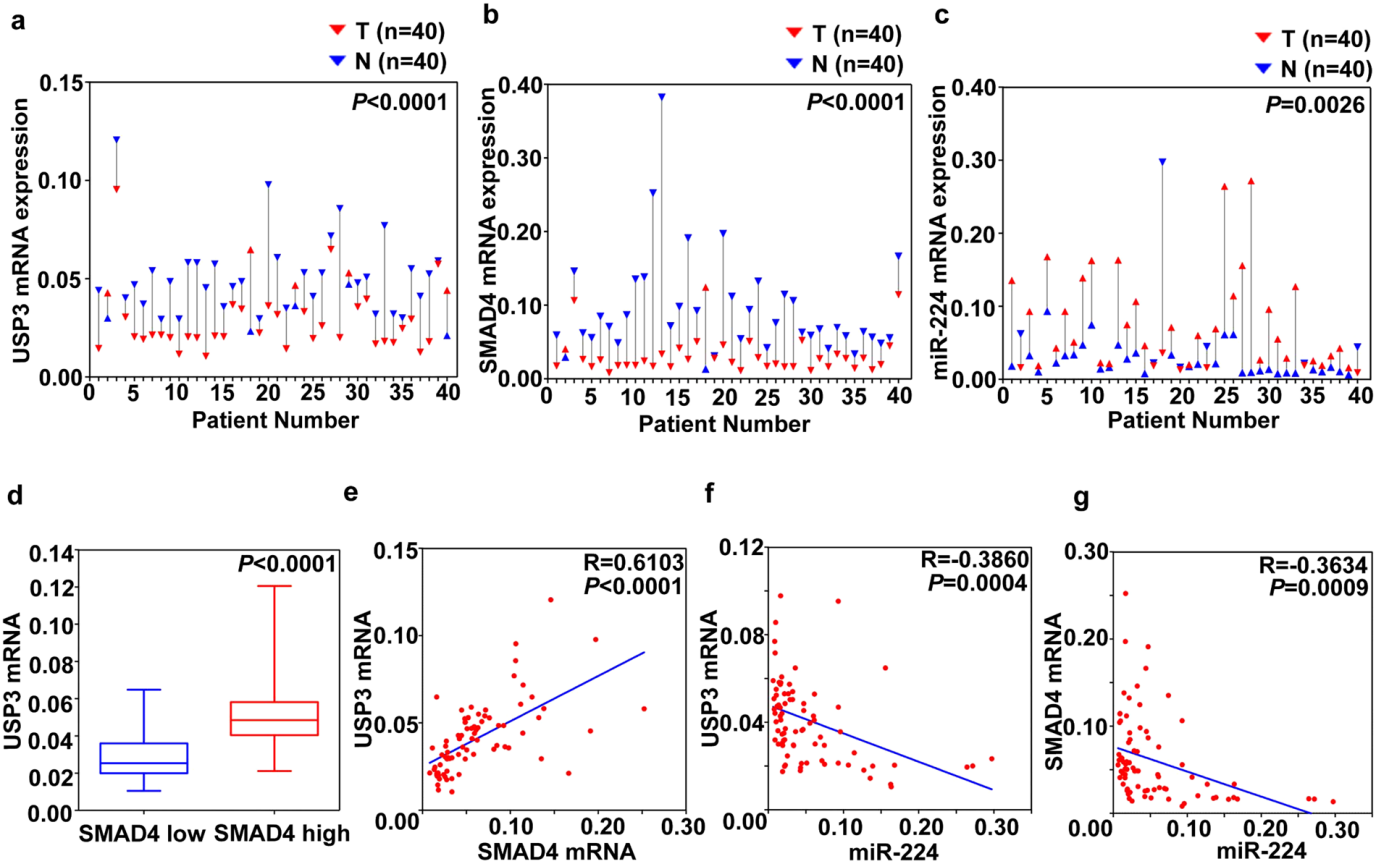
Expression and correlation of USP3, SMAD4 and miR-224 in primary CRC samples (**a–c**) USP3, SMAD4 and miR-224 expression in 40 paired primary CRC and adjacent non-cancer tissues were evaluated by qRT-PCR. (**d**) Comparison of the USP3 expression levels between “SMAD4 low” and “SMAD4 high” subgroups that were classified according to the median SMAD4 expression levels. (**e–g**) Correlation analyses of USP3 mRNA, SMAD4 mRNA and miR-224 were conducted in these CRC specimens.

To verify whether USP3 regulated SMAD4 through miR-224 *in vivo*, we evaluated their expression in 40 pairs of human CRC and matched noncancerous specimens. The USP3 and SMAD4 mRNAs were downregulated in CRC compared with matched normal tissues (Fig. 5a,b). Meanwhile, miR-224 expression was increased in the CRC tissues (Fig. 5c). The expression of USP3 was significantly correlated with SMAD4 transcript levels when samples were subdivided into “SMAD4 low” and “SMAD4 high” groups (Fig. 5d). Furthermore, the Pearson coefficient represented a significant positive correlation between SMAD4 and USP3 (Pearson R = 0.6103, *P* < 0.0001) (Fig. 5e). Consistently, an inverse correlation was found not only between miR-224 and USP3 (Pearson R = −0.3860, *P* = 0.0004) (Fig. 5f) but also between miR-224 and SMAD4 (Pearson R = −0.3634, *P* = 0.0009) (Fig. 5g). The correlation of USP3, SMAD4 and miR-224 was also verified in GSE29623, a GEO dataset containing mRNA and miRNA expression profiles of 65 colon cancer specimens (Supplementary Fig. S5).

### Expression of USP3 mRNA and its correlation with clinicopathological characteristics in patients with CRC

**Figure 6:**
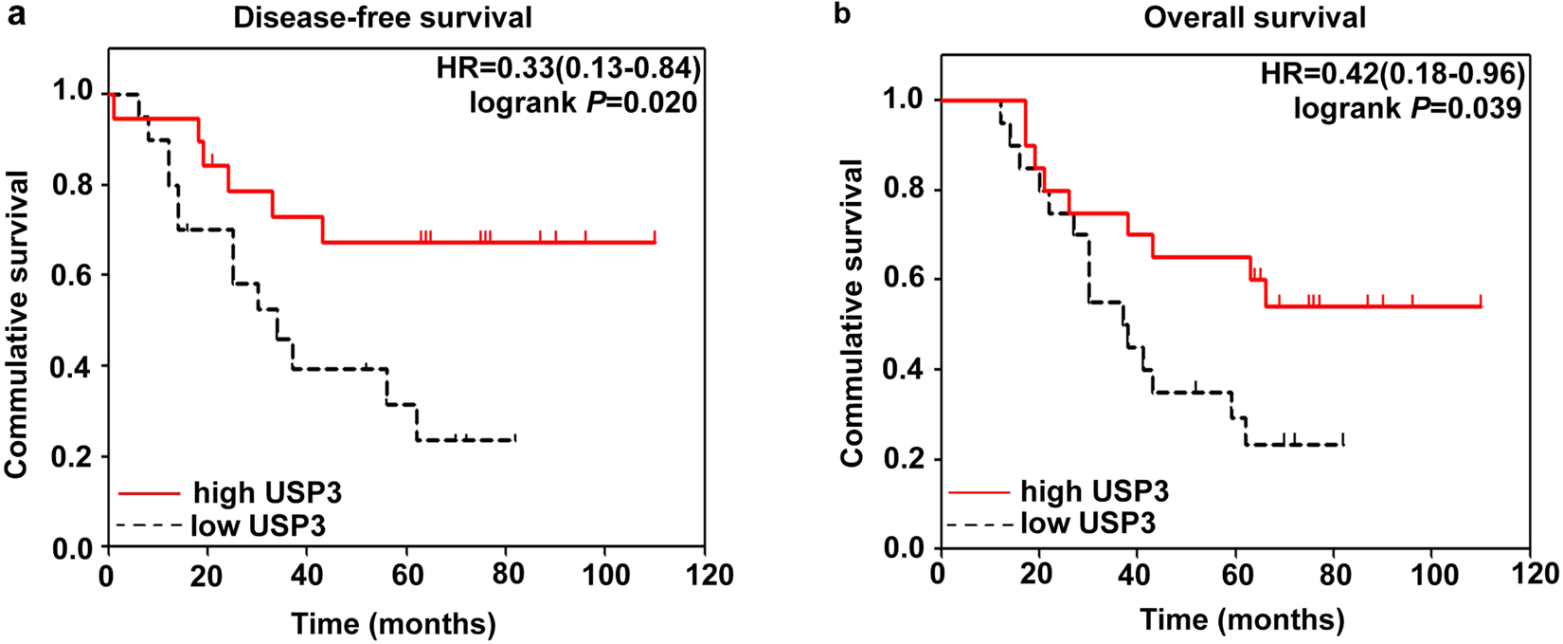
Kaplan-Meier curves for the disease-free survival and overall survival of the 40 CRC patients. High USP3 mRNA expression was notably correlated with favourable DFS (**a**) and OS (**b**) in the CRC patients.

To determine the clinical significance of USP3, we investigated the relationship between its expression and the clinicopathological features of the patients involved in our study. As shown in Table 1, USP3 mRNA level was significantly associated with distal metastasis (*P* = 0.023), but were not correlated with other factors. What’s more, patients with low USP3 expression had significantly shorter disease-free survival (DFS, *P* = 0.02, Fig. 6a) and overall survival (OS, *P* = 0.039, Fig. 6b) than those with high USP3 expression. The results indicated that downregulated USP3 mRNA level was correlated with a worse prognosis in CRC patients.

**Table 1:** Association between the expression of USP3 and clinicopathological features in 40 patients with CRC. *P < 0.05.

Factors	No. of patients (%)	USP3 (2^−ΔΔCt^) Mean ± SD	*P* value
Gender			
Male	30 (75.0%)	0.225 ± 0.021	0.900
Female	10 (25.0%)	0.219 ± 0.029	
Age (yr)			
≥60	20 (50.0%)	0.219 ± 0.025	0.806
<60	20 (50.0%)	0.228 ± 0.025	
Tumour size (cm)			
≥5	17 (42.5%)	0.238 ± 0.030	0.468
<5	23 (57.5%)	0.212 ± 0.020	
Depth of invasion			
T2 + T3	13 (32.5%)	0.235 ± 0.039	0.640
T4	27 (67.5%)	0.218 ± 0.018	
Lymphatic metastasis			
Positive	26 (65.0%)	0.242 ± 0.022	0.140
Negative	14 (35.0%)	0.189 ± 0.025	
Distal metastasis			
Positive	20 (50.0%)	0.185 ± 0.018	0.023*
Negative	20 (50.0%)	0.262 ± 0.027	
Stage (AJCC)			
Stage II	14 (35.0%)	0.186 ± 0.025	0.140
Stage III/IV	26 (65.0%)	0.242 ± 0.022

**Figure 7:**
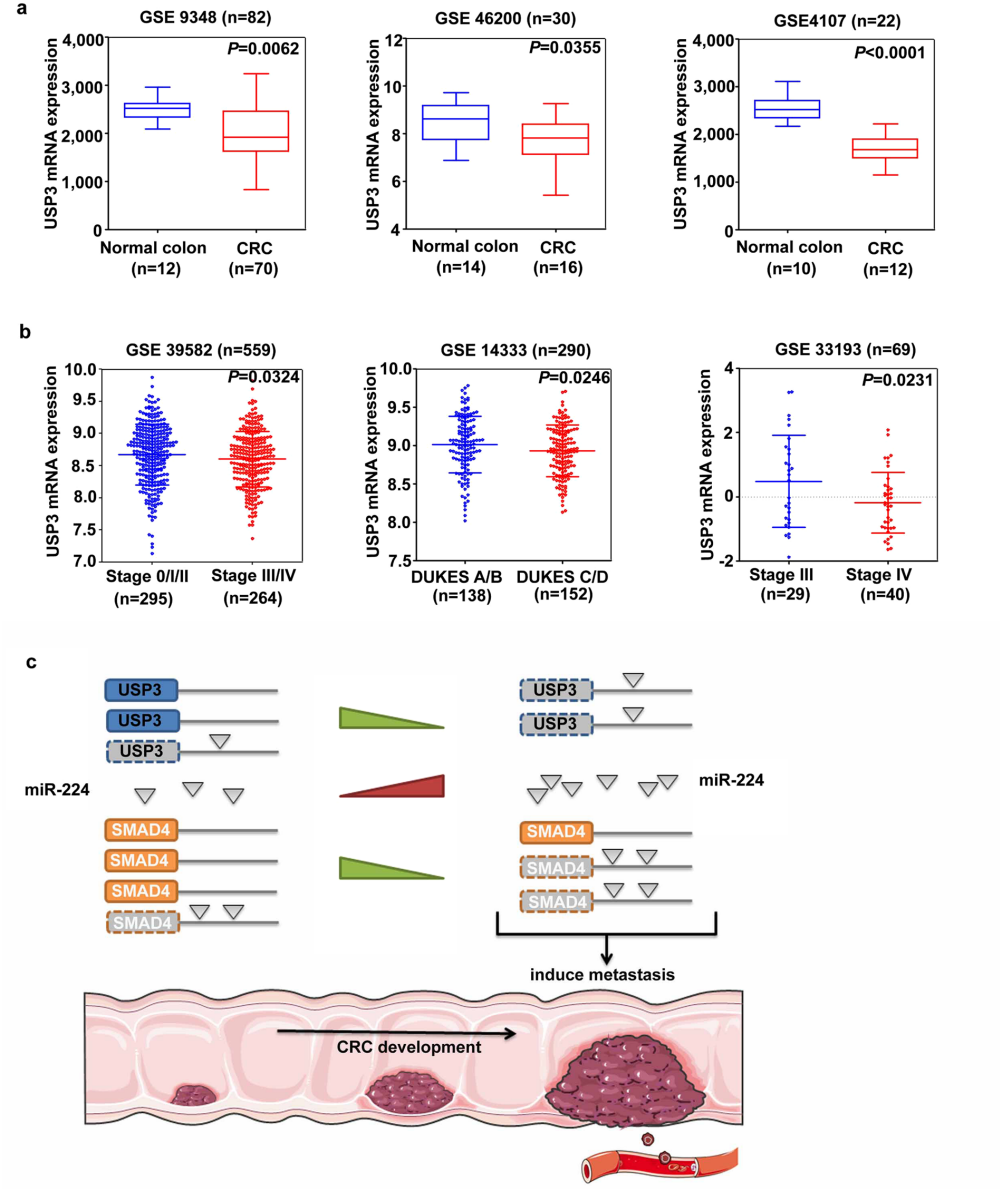
Analyses of USP3 mRNA expression in the CRC samples from the GEO database. (**a**) USP3 mRNA was decreased in CRCs compared with their adjacent noncancerous tissues (GEO datasets: GSE9348, GSE46200 and GSE4107). (**b**) Reduced expression of USP3 mRNA was associated with an advanced tumour stage in the primary CRC tissues (GEO datasets: GSE39582, GSE14333 and GSE33193). (**c**) A schematic model illustrates how a dysregulated USP3-miR-224-SMAD4 axis facilitates CRC metastasis during cancer development.

In addition to our findings that USP3 mRNA was significantly downregulated in CRC tissues, data from three GEO datasets (GSE9348, GSE46200 and GSE4107), which contained information of USP3 expression in the normal mucosa and CRC tissues, provided further validation (Fig. 7a). Besides, we observed that reduced USP3 expression was remarkably correlated with advanced tumour stage in three other CRC datasets (GSE39582, GSE14333 and GSE33193) (Fig. 7b).

## Discussion

SMAD4, a critical mediator of the TGF-β signalling pathway, is frequently inactivated due to chromosomal instability or intragenic mutations that occur in the development of CRC^4^. Loss of SMAD4 contributes to the disruption of TGF-β signalling^3, 4, 40^ and is also indicative of advanced tumour stage and a worse prognosis^7–11^. Nevertheless, the sensitivities of the 18q21 allelic imbalance and SMAD4 mutations as prognostic markers are not as high as that of SMAD4 protein level^41^. What’s more, There is no difference in SMAD4 protein levels between tumours with 18q21 allelic imbalance and those without allelic imbalance^41^. Therefore, it will be important to elucidate the additional mechanisms that modulate SMAD4 expression. Recent studies have demonstrated that SMAD4 is also regulated by miRNAs^13–17^. These miRNAs do not work alone, but are instead involved in a more complicated RNA communication network, which is known as ceRNA crosstalk. Through sponging common miRNAs shared by other transcripts, ceRNAs could regulate one another more subtlety^23–25^. To date, ceRNAs for SMAD4 remains unknown.

In the present study, we found that as a protein-coding mRNA, USP3 communicated with SMAD4 in a miRNA and 3′UTR dependent manner. USP3 and SMAD4 crosstalked with each other at the post-transcriptional level, and both were direct targets of miR-224. Their mRNAs were negatively correlated with miR-224 in CRC specimens. In addition, attenuated USP3 expression facilitated CRC cell migration and invasion, while overexpression of USP3 3′UTR reversed this malignant phenotype. This reversal was partially due to the ceRNA interaction between USP3 and SMAD4. Moreover, decreased USP3 mRNA expression in primary CRC has been associated with advanced tumour stage and distal metastasis. Additionally, lower USP3 mRNA expression predicted a worse prognosis not only for CRC patients, but also for patients with other malignancies, such as gastric cancer, lung cancer and breast cancer (Supplementary Fig. S6). These suggested an important role for USP3 in tumourigenesis.

USP3 belongs to the DUB protein family, which is a group of enzymes that participate in the reverse process of ubiquitination by removing ubiquitin from target proteins and rescuing proteins that are marked for degradation^42^. USP3 has been demonstrated to be an important modifier of chromatin to maintain genome integrity by deubiquitinating H2A/H2B. Ablation of USP3 attributes to delayed S phase progression and the accumulation of DNA breaks^43^. USP3-deficient mice present significantly shorter life spans and an increased haematopoietic cancer incidence^44^. Interestingly, SMAD4 is not subjected to ubiquitin-mediated degradation^45^. Nevertheless, the impact of USP3 on cancer development has not previously been reported. In this study, we demonstrated that decreased USP3 mRNA facilitated CRC progression by reducing SMAD4 protein level in a miR-224 dependent manner. This mechanism illustrated the complexity of epigenetic modifications by RNA-based regulation.

Recently, the 3′UTRs of protein-coding genes have been proven to be important molecular players in cancer biology through *cis* or *trans* modulation of gene expression^46, 47^. *Cis-*acting roles of 3′UTRs refer to regulatory effects that focus on its own protein-coding genes, while *trans*-acting effects indicate functions that are exerted beyond its corresponding transcripts. The ceRNA interaction with the 3′UTR belongs to the latter^46^. In this study, we have confirmed that the USP3 3′UTR acts as a *trans* modulator of SMAD4. Although elevated protein expression was not observed when the USP3 or SMAD4 3′UTRs were overexpressed alone, the protein levels increased when USP3 and SMAD4 were first silenced, indicating that the ceRNA crosstalk between them only occurred when the transcript quantities dropped to relatively low levels. The dynamics and constraints of ceRNA crosstalk may contribute to this partially effective ceRNA regulation^22, 48–50^. ceRNA interactions have been reported to occur among a small subset of transcripts whose amount and corresponding miRNA amount have fell within a narrow range. Additionally, the optimal abundance of miRNAs and ceRNAs within a network should near equimolarity^22^. If the ceRNA transcript abundance overwhelmingly exceeds that of the miRNAs, the ceRNA crosstalk would be minimized because the miRNAs are occupied at any given time and are unlikely to participate in gene regulation^22, 48^. When endogenous USP3 and SMAD4 mRNA are knocked down, limited miR-224 is released to subsequently exert its function. During the development of CRC, elevated miR-224 expression was correlated with advanced tumour stage^13–15^, while the expression of USP3 and SMAD4 transcripts were reduced with respect to tumour stages. As the schematic model shown in Fig. 7c, we propose that with the progression of CRC, decreased USP3 mRNA releases more unbound miR-224, to suppress SMAD4 expression and to accelerate tumour progression.

There are some limitations of the present study. Although 22 sponge miRNAs were predicted to be shared by USP3 and SMAD4 (Supplementary Table S2), we focused on miR-224 due to its highest mirSVR score. Further study is required to validate whether the other 21 predicted miRNAs participate in communications between SMAD4 and USP3 and to shed light on the complete ceRNA network between them.

In conclusion, our data demonstrated that, as a direct target of miR-224, USP3 is a potent ceRNA of SMAD4 in CRC. Decreased expression of USP3 mRNA is associated with a poor prognosis and distal metastasis of CRC, which is attributed to further suppression of SMAD4 through the release of miR-224 from the USP3 3′UTR. As a sponge of miR-224, the USP3 3′UTR may be a promising miR-224 inhibitor that could be applied in CRC gene therapy to inhibit tumour progression in the near future.

## Methods

### Ethics Statement

The methods in this study were carried out in accordance with the approved guidelines. All experimental protocols were approved by the Research Ethics Committee of Peking University Cancer Hospital & Institute.

### Patients and Tissue Specimens

Paired CRC and adjacent normal tissues were collected from 40 patients who underwent operations at Peking University Cancer Hospital & Institute from 2004 to 2007. The tissues were fresh-frozen and stored at −80 °C. None of patients involved in this study had previously been treated with adjuvant chemotherapy, radiotherapy or immunotherapy. TNM staging of the patients was carried out based on the American Joint Committee on Cancer (AJCC, USA) staging criteria. The clinicopathological characteristics of the patients are summarized in Table 1. The median follow-up period was 55 months. This project was approved by the Research Ethics Committee of Peking University Cancer Hospital & Institute. Written informed consents were obtained from all patients prior to surgery according to the guidelines.

### Cell Lines and Cell Culture

LoVo, HCT116, SW480 and RKO human CRC cell lines and HEK293T cells were purchased from the American Type Culture Collection. Cells were conventionally maintained in DMEM or RPMI-1640 (Gibco, Carlsbad, CA, USA) medium supplemented with 10% foetal bovine serum in a humidified atmosphere with 5% CO_2_ at 37 °C.

### Plasmid, siRNA, shRNA-containing lentivirus, miRNA mimics, as well as inhibitors and transfection

To overexpress USP3 and SMAD4, their coding sequences were inserted into pcDNA3.1 vector (P3.1 for short), and the constructs (P3.1-USP3 CDS and P3.1-SMAD4 CDS) were verified by DNA sequencing. Expression plasmids containing the full-length 3′UTR of USP3 and SMAD4 (P3.1-USP3 3′UTR and P3.1-SMAD4 3′UTR) were synthesized by Sangon (Shanghai). The SMAD4 and USP3 3′UTR were then subcloned into a luciferase reporter vector (PGL3-control, Promega) and were denoted as PGL3-USP3 3′UTR and PGL3-SMAD4 3′UTR, respectively. Additionally, the miR-224-targeting sequences of the USP3 and SMAD4 3′UTRs were inserted into PGL3-control vector (PGL3-WT-USP3 and PGL3-WT-SMAD4). Mutant constructs with the deleted sequence of the core binding site of miR-224 (PGL3-MUT-USP3, PGL3-MUT1-SMAD4, PGL3-MUT2-SMAD4 and PGL3-Double-MUT) were generated by TaKaRa MutanBEST Kit (TaKaRa, Japan). The primers used are summarized in Supplementary Table S1.

The USP3-targeting siRNAs (SI00089432, SI00089439, SI00089446, SI03071432), SMAD4-targeting siRNAs (SI00076041, SI03089527), Dicer-targeting siRNAs (SI02655492, SI00300006), and a universal negative control siRNA (si-NC) were purchased from QIAGEN (Hilden, Germany). Lentiviruses that expressed an shRNA vector that targeted USP3 and a control vector were constructed, packaged, and purified by GenePharma Corporation (Shanghai, China). MiR-224 mimics/inhibitors and the negative control (mi-NC) were synthesized by Invitrogen.

Plasmids, siRNAs, miR-224 mimics/inhibitors, and their negative controls were transiently delivered to cells using Lipofectamine™ 3000 (Invitrogen, Carlsbad, CA).

### Western Blot

Cells were lysed in RIPA buffer containing a protease inhibitor. Equal amounts of protein were subjected to SDS-PAGE and transferred to nitrocellulose membranes (Millipore). The membranes were incubated overnight with a primary antibody against USP3 (1:200 dilution, ab101473, Abcam, USA), SMAD4 (1:200 dilution, Smad4(B-8), Santa Cruz, USA) or β-actin (1:3000 dilution, AC-15, Sigma, USA). Afterwards, goat anti-mouse IgG or goat anti-rabbit IgG (ZSGB-BIO) was used as a secondary antibody. An ECL detection kit (Amersham, Little Chalfont, UK) was used to detect the signal. The ImageJ software (NIH, USA) was used for quantification.

### Quantitative Real-time PCR (qRT-PCR)

Total RNA from cells and tissues was extracted using the TRIzol reagent (Invitrogen, Carlsbad, CA, USA). The Reverse Transcription Kit (Promega, Madison, WI, USA) was used to perform reverse transcription for cDNA. After mixing the SYBR Green PCR Master Mix (Toyobo, Osaka, Japan) with the primers listed in Supplementary Table S1, qRT-PCR was performed using the ABI 7500 Real-time PCR System (Life Technologies, Carlsbad, California, USA). TaqMan MicroRNA Assays were used to detect miR-224 expression. GAPDH or U6 were used as internal controls.

### Luciferase Reporter Assay

Cultured cells were transfected with the indicated luciferase reporter plasmids (200 ng), miR224 mimics/mi-NC (100 nM), and the internal control plasmid (pRL-SV40, 10 ng). Luciferase activity was determined 48 h after transfection using the dual-luciferase reporter assay system (Promega). Firefly luciferase activity was normalized to the Renilla luciferase activity.

### Wound-healing Assays

LoVo and HCT116 cells were transfected with corresponding plasmids or siRNAs in 6-well plates and cultured to near total confluence. Wounds were produced in the cell monolayer using a sterile 200-µl pipette tip. After cells were rinsed with PBS, serum-free DMEM was added to the media. Images were obtained from three separate wounds at 0 and 24 h post-scratch, and the distances between the two edges were measured.

### Transwell Migration/Invasion Assays

Migration assays were performed using a Boyden chamber that contained a polycarbonate filter with an 8-µm pore size (Costar). Serum-free DMEM medium was added to the upper chamber, while complete medium containing 10% FBS was added to the lower chamber. After transfection, 8 × 10^4^ LoVo and 2 × 10^5^ HCT116 cells were separately seeded in the upper chambers and incubated for 48 h. The migrated cells were stained with crystal violet, and five random fields were captured by microscopy. Cell invasion was similarly detected except that the upper chamber was pre-coated with Matrigel (5 mg/ml, BD Biosciences).

### *In vivo* Metastasis

All experiments were approved by the Animal Ethics Committee of Peking University Cancer Hospital & Institute and performed in full compliance with the Experimental Animal Management Ordinance. Female NOD/SCID mice (20 g) were purchased from Hua-Fu-Kang Corporation (Beijing, China). Each mouse was injected via the tail vein with 2 × 10^6^ LoVo cells that stably expressed sh-USP3 or the control sh-NC vector. Animals were sacrificed 8 weeks after injection. The lungs from each mouse were isolated, fixed with 4% paraformaldehyde, and stained with 3% picric acid, followed by paraffin-embedding and haematoxylin and eosin staining for histological analysis.

### MicroRNA Target Prediction

Four microRNA target prediction algorithms — TargetScan (http://www.targetscan.org/), miRanda (http://www.microrna.org/), rna22 (https://cm.jefferson.edu/rna22/) and Starbase V2.0 (http://starbase.sysu.edu.cn/) — were used to obtain miRNA binding sites in the SMAD4 3′UTR or candidate ceRNAs. The Oncomine database (http://www.oncomine.org) was used to identify differentially expressed genes in CRC, and the mentha database (http://mentha.uniroma2.it/) was used to clarify whether a direct interaction existed between the USP3 and the SMAD4 proteins.

### Statistical Analysis

All experiments were performed at least three times with each sample tested in triplicate. Data are displayed as the mean ± standard deviation (SD). The difference between two groups was calculated by two-tailed Student’s t-test using SPSS software 20.0 (SPSS, Chicago, IL) and GraphPad Prism 5 software (GraphPad, San Diego, USA). The Kolmogorov-Smirnov test was performed to determine whether samples were normally distributed. Differences between expression levels of USP3, SMAD4 and miR-224 in CRC tissues and adjacent noncancerous tissues were analysed by paired t-test. Pearson correlation analysis was conducted to evaluate the association between USP3, SMAD4 and miR-224. Survival was estimated using the Kaplan-Meier method, and the statistical differences were estimated by the log-rank test. *P* < 0.05(*) was defined as statistically significant.

### Data Availability

All data generated or analysed during this study are included in this published article (and its Supplementary Information files).

## Electronic supplementary material

Supplementary Information
