# Supplementary material for: Downregulated USP3 mRNA functions as a competitive endogenous RNA of SMAD4 by sponging miR-224 and promotes metastasis in colorectal cancer
Source: Sci Rep. 2017 Jun 27;7:4281. doi: 10.1038/s41598-017-04368-3 (PMC5487320; doi:10.1038/s41598-017-04368-3)
Supplement: Supplementary file 1 — Supplementary Information [file 41598_2017_4368_MOESM1_ESM.pdf]

Supplementary Information for

Downregulated USP3 mRNA functions as a competitive endogenous RNA of SMAD4 by sponging miR-224 and promotes metastasis in colorectal cancer

Zaozao Wang[1†], Jie Yang[1†], Jiabo Di[1], Ming Cui[1], Jiadi Xing[1], Fan Wu[1], Wei Wu[1], Hong Yang[1], Chenghai Zhang[1], Zhendan Yao[1], Nan Zhang[1], Beihai Jiang[1]*, Xiangqian Su[1]*

[1]Key Laboratory of Carcinogenesis and Translational Research (Ministry of Education), Department Gastrointestinal Surgery IV, Peking University Cancer Hospital & Institute, Beijing 100142, China.

[†]These authors contributed equally to this work.

*Corresponding authors.

Correspondence to:

Xiangqian Su

Key Laboratory of Carcinogenesis and Translational Research (Ministry of Education)

Department Gastrointestinal Surgery IV

Peking University Cancer Hospital & Institute

52 Fucheng Road, Haidian District, Beijing 100142, China

Tel.: 8610-88196579; Fax: 8610-88122437

E-mail: suxiangqian@bjmu.edu.cn

Beihai Jiang

Key Laboratory of Carcinogenesis and Translational Research (Ministry of Education)

Department Gastrointestinal Surgery IV

Peking University Cancer Hospital & Institute

52 Fucheng Road, Haidian District, Beijing 100142, China

Tel.: 8610-88196836; Fax: 8610-88122437

E-mail: beihai_jiang@bjmu.edu.cn

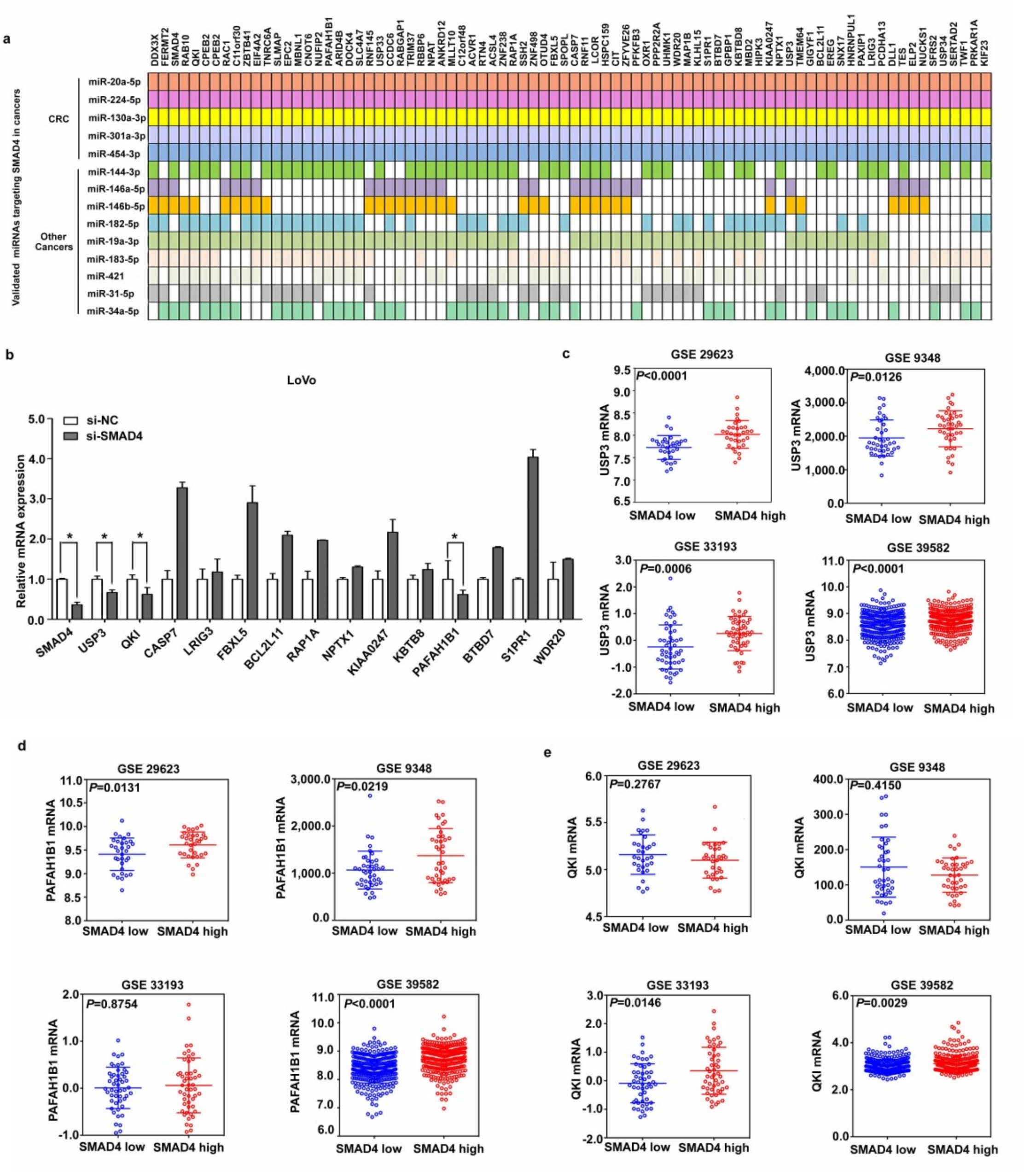

Supplementary Figure S1

Identification of putative SMAD4 ceRNAs in CRC.

(a) Bioinformatics approaches were applied to identify putative SMAD4 ceRNAs in CRC. Genes that shared eight or more common MREs with SMAD4 were considered as SMAD4 ceRNAs candidates. (b) mRNA expression of 14 putative SMAD4 ceRNAs were examined after siRNA mediated knockdown of SMAD4 by qRT-PCR. (c) USP3 mRNA was correlated with SMAD4 in four GEO datasets of CRC. (d) PAFAH1B1 or (e)

QKI mRNA was correlated with SMAD4 in either three or two GEO datasets of CRC, respectively.

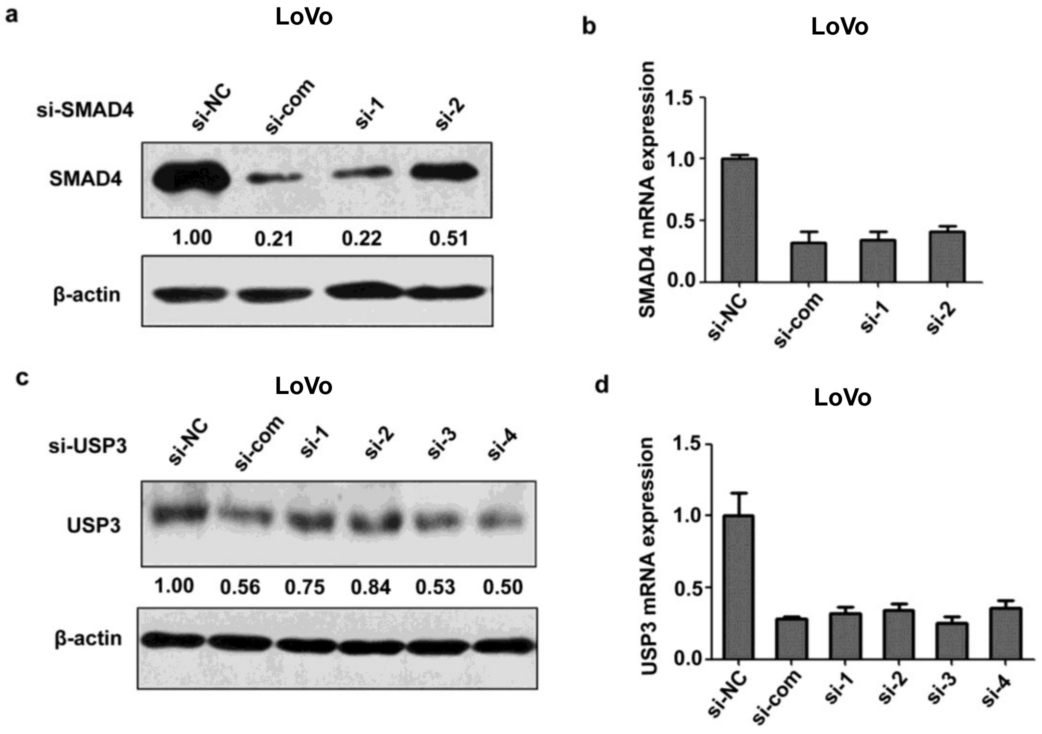

Supplementary Figure S2

Efficiency of siRNAs-mediated knockdown of SMAD4 and USP3 in LoVo cells.

After transfection of SMAD4 siRNAs or USP3 siRNAs with their negative control, protein and mRNA expressions of SMAD4 and USP3 were examined by western blot (a,c) and qRT-PCR (b,d). Figures of western blot were cropped and full-length blots were included in Supplementary Fig. S17 and Fig. S18. Quantitative protein expression data were normalized to β-actin. * $P<0.05$.

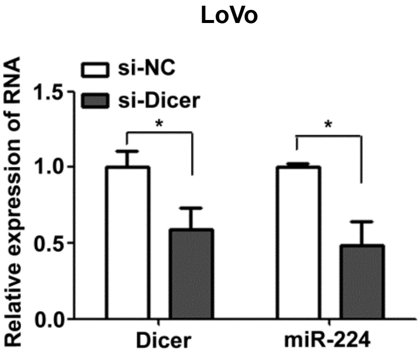

Supplementary Figure S3

Transfection efficiency of Dicer siRNAs and its effect on miRNA expression.

After transfection of Dicer siRNAs with its negative control, Dicer mRNA expression was detected by qRT-PCR, along with silencing of Dicer, miRNA expression was also decreased (miR-224 was detected as an example).

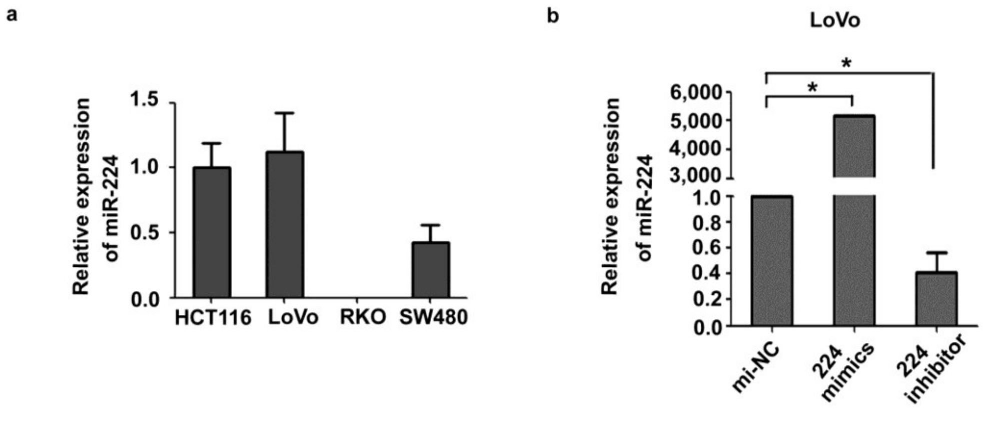

Supplementary Figure S4

qRT-PCR was utilized to detect (a) the miR-224 expression in CRC cell lines and (b) the transfection efficiency of miR-224 mimics/inhibitor in LoVo cells.

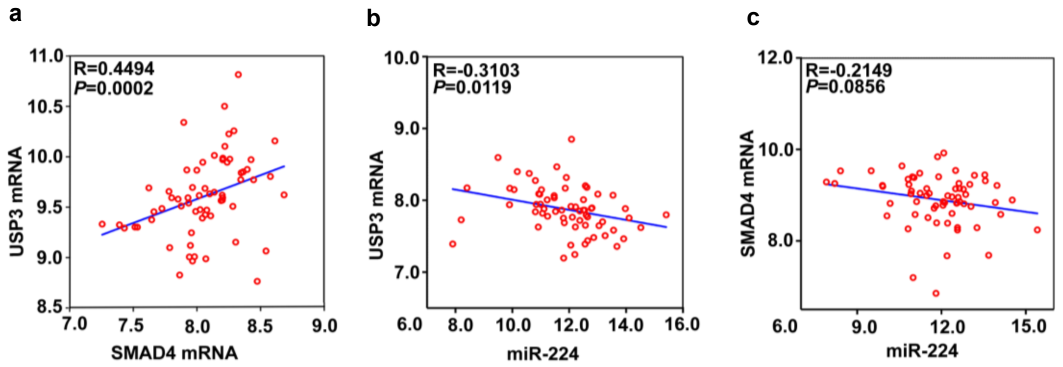

Supplementary Figure S5

Correlation of USP3, SMAD4 and miR-224 in GSE29623, a GEO dataset containing mRNA and miRNA expression profiles of 65 colon cancer specimens.

(a) USP3 and SMAD4 mRNA expression showed a positive and significant correlation. (b) USP3 mRNA was negatively correlated with miR-224 while (c) the correlation between SMAD4 mRNA and miR-224 was not so obvious in this dataset.

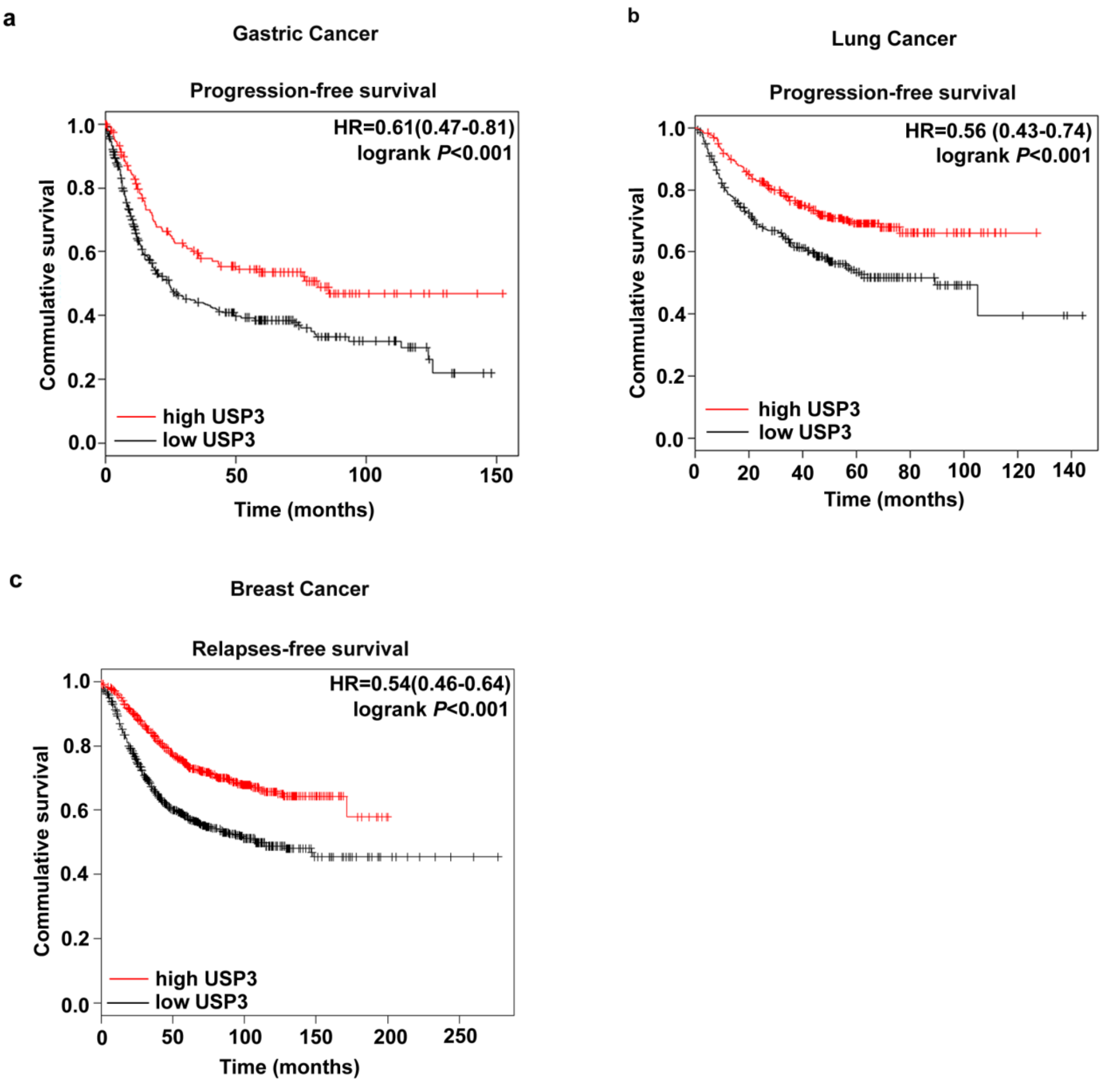

Supplementary Figure S6

Kaplan-Meier estimates for progression-free survival and relapse-free survival of patients with high or low USP3 mRNA expression in a published microarray dataset (The KM Plotter tool which is short of CRC data).

Kaplan-Meier curves based on analysis of a published microarray dataset from (a) gastric cancer, (b) lung cancer and (c) breast cancer patients showed progression-free survival and relapse-free survival of patients separated into high and low USP3 mRNA expression.

Figure S7 to Figure S18 displayed full-length blots of cropped WB results used in the main figures.

There were two points we would like to mention:

1. Antibody against USP3 (Abcam, ab101473) was a rabbit polyclonal antibody, so that there often appeared 2 to 3 non-specific bands between 110-70 kDa, while antibodies against SMAD4 (Santa Cruz, B-8) and β-actin (Sigma, AC-15) were mouse monoclonal antibodies, therefore the bands detected are unique.
2. In order to save reagents, sometimes we arranged different nitrocellulose membranes closely and exposed them together. Therefore, some irrelevant bands of other experiments may be detected in the same uncropped blot. And we indicated them in corresponding figures.

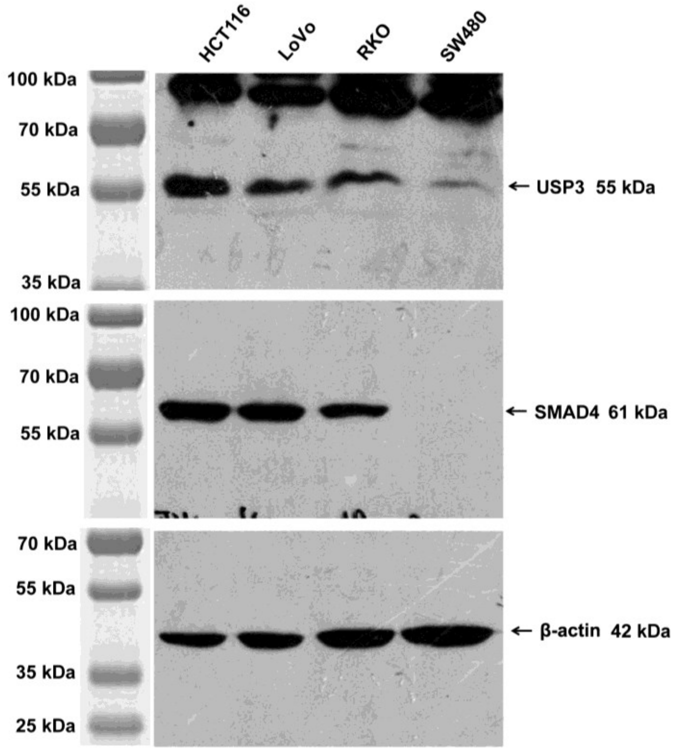

Supplementary Figure S7

Uncropped blots probed with USP3, SMAD4 and β-actin in Figure 1a.

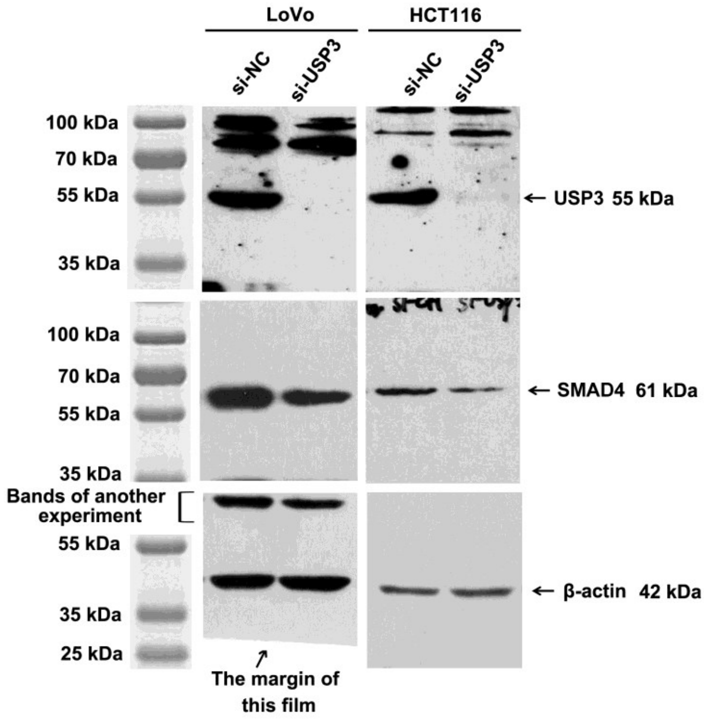

Supplementary Figure S8

Uncropped blots probed with USP3, SMAD4 and β-actin in Figure 1c.

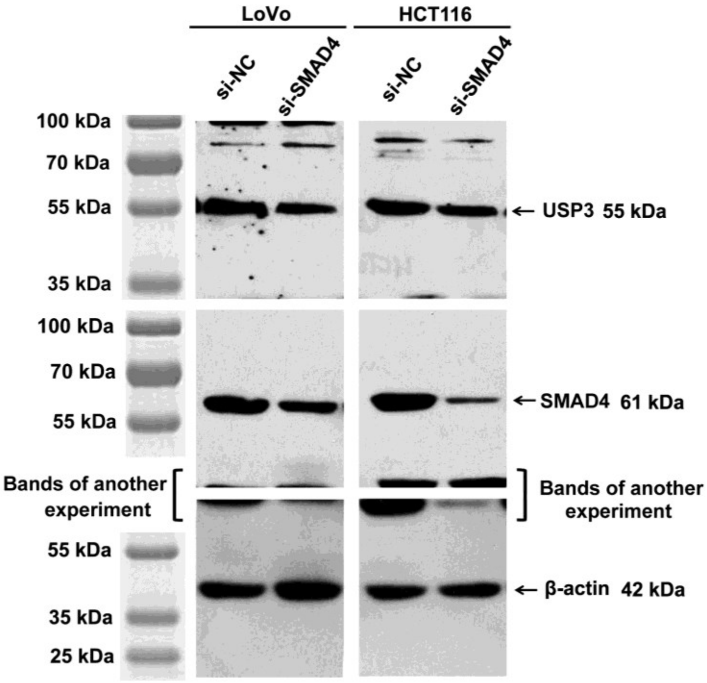

Supplementary Figure S9

Uncropped blots probed with USP3, SMAD4 and β-actin in Figure 1e.

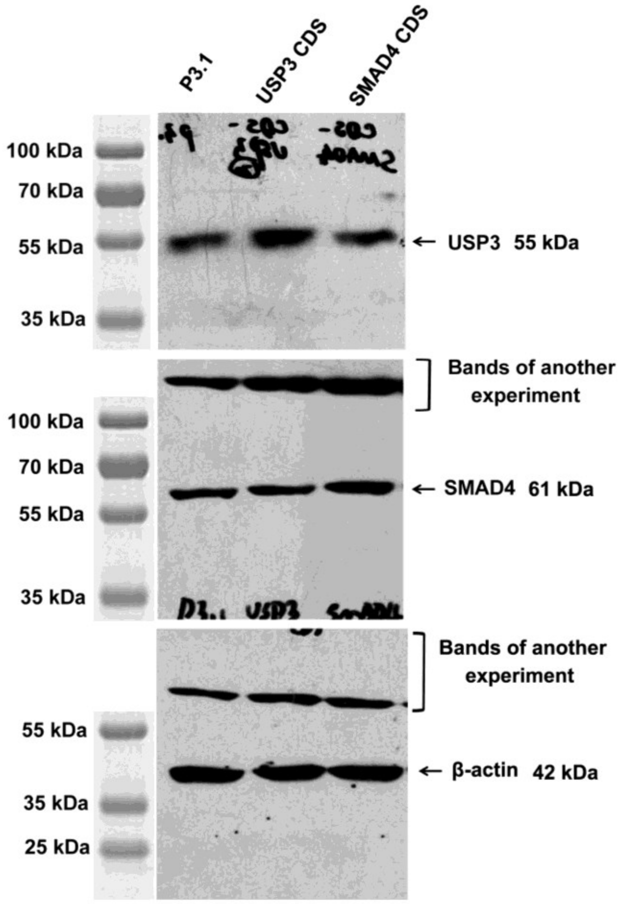

Supplementary Figure S10

Uncropped blots probed with USP3, SMAD4 and β-actin in Figure 1g.

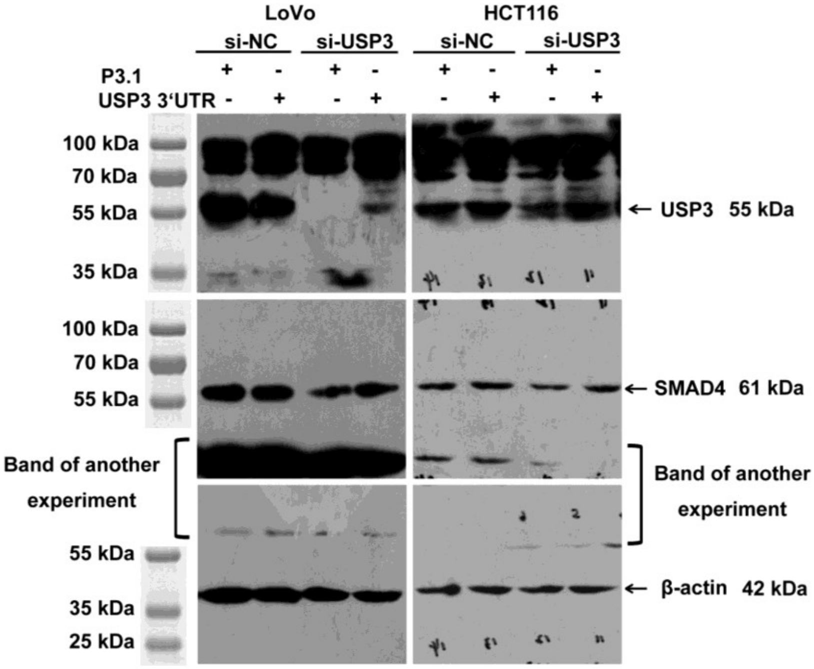

Supplementary Figure S11

Uncropped blots probed with USP3, SMAD4 and β-actin in Figure 2a.

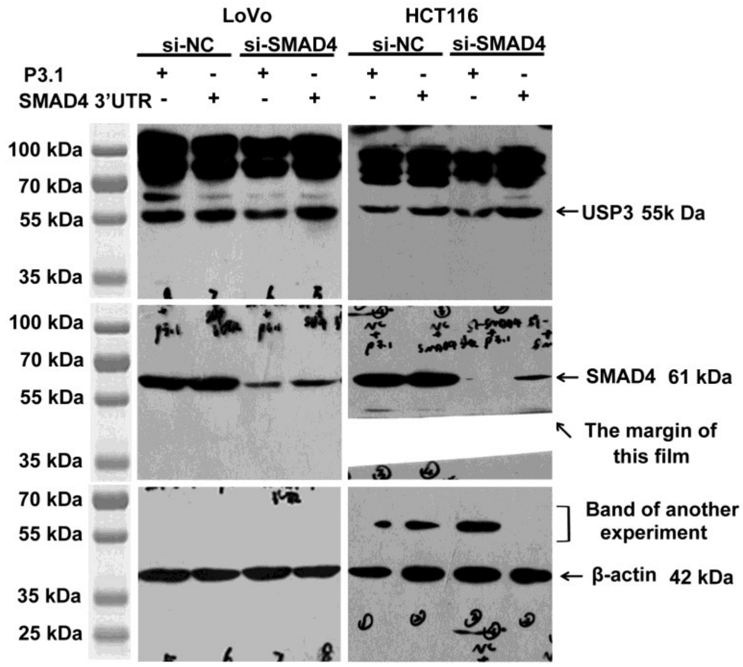

Supplementary Figure S12

Uncropped blots probed with USP3, SMAD4 and β-actin in Figure 2b.

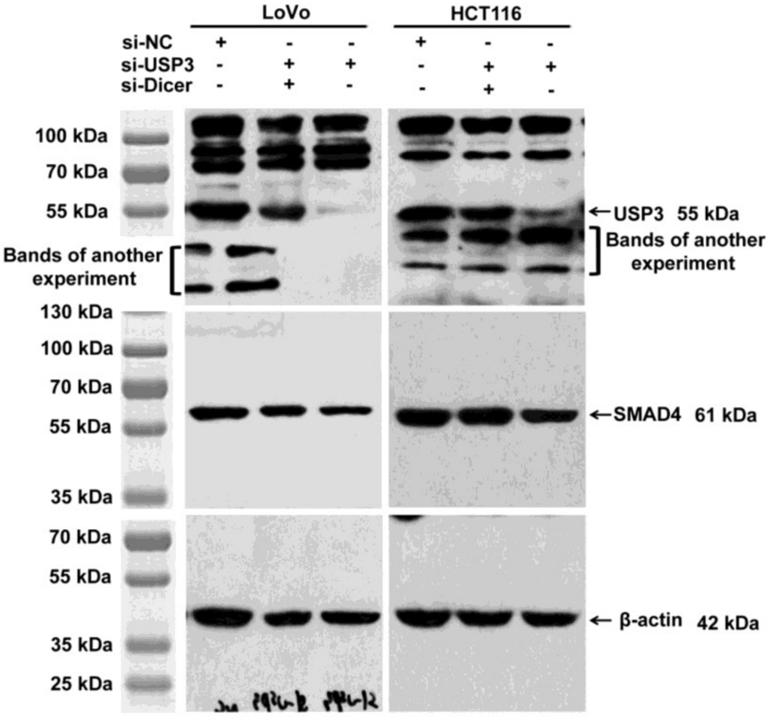

Supplementary Figure S13

Uncropped blots probed with USP3, SMAD4 and β-actin in Figure 2g.

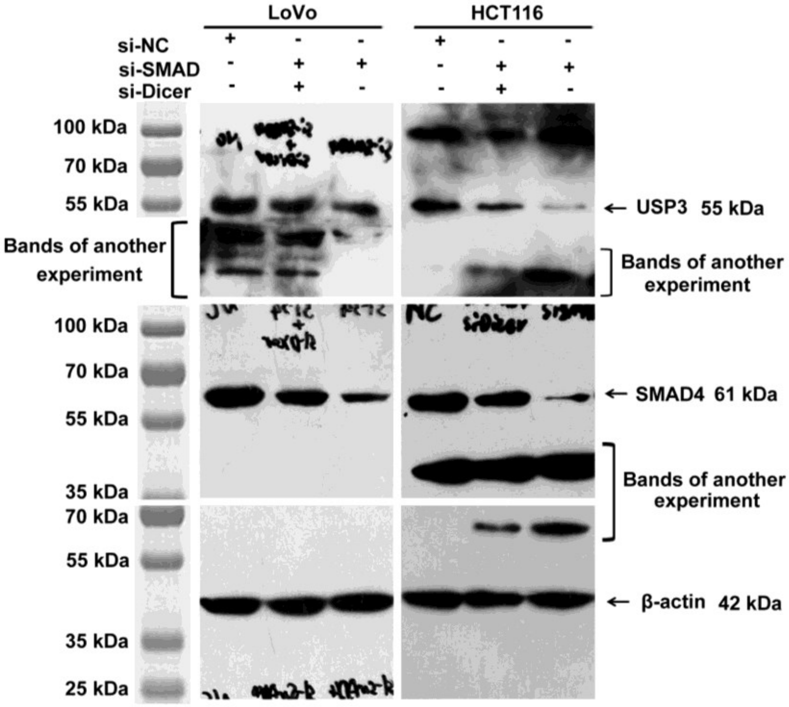

Supplementary Figure S14

Uncropped blots probed with USP3, SMAD4 and β-actin in Figure 2h.

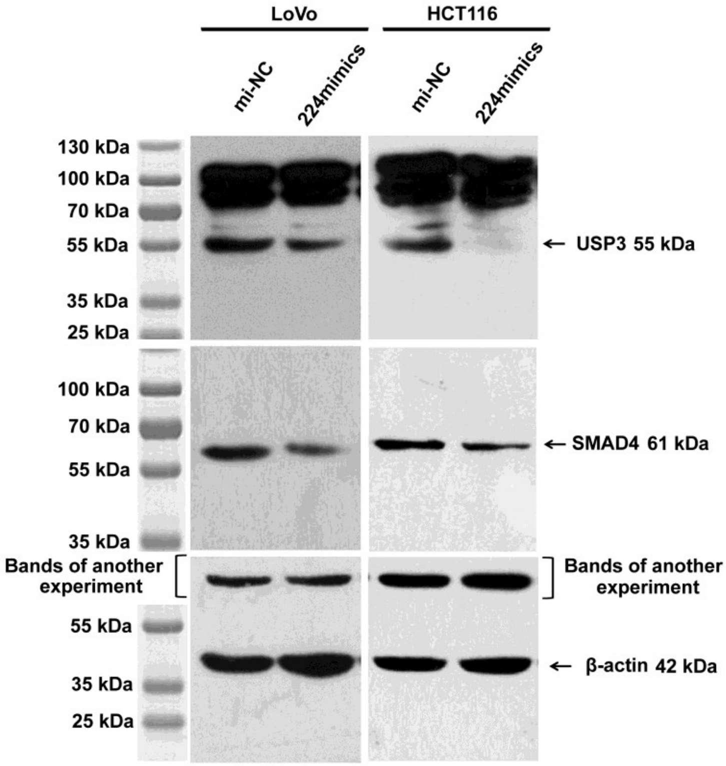

Supplementary Figure S15

Uncropped blots probed with USP3, SMAD4 and β-actin in Figure 3a.

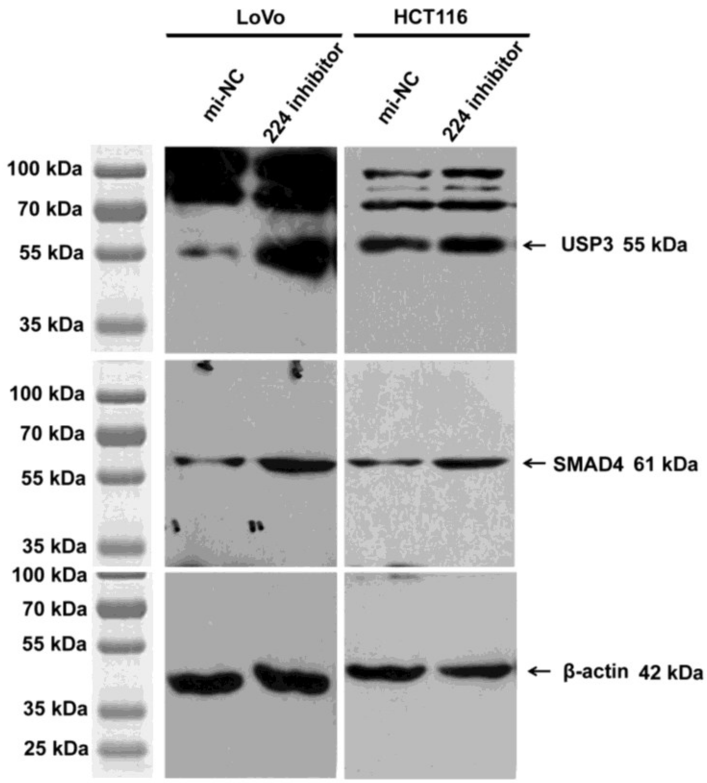

Supplementary Figure S16

Uncropped blots probed with USP3, SMAD4 and β-actin in Figure 3b.

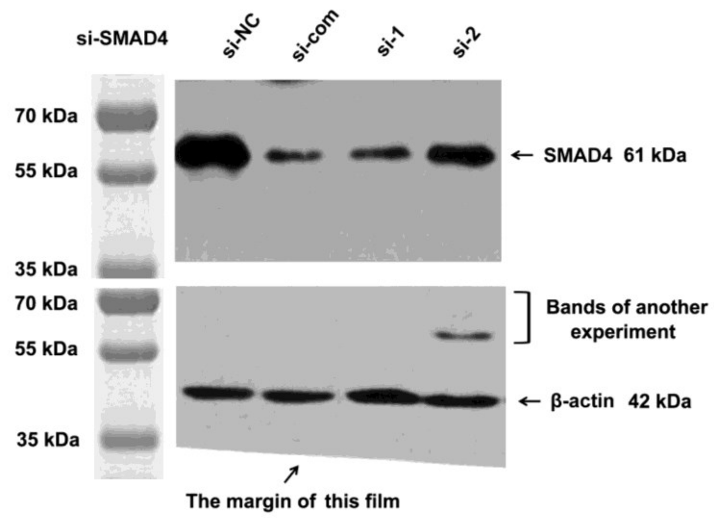

Supplementary Figure S17

Uncropped blots probed with USP3, SMAD4 and β-actin in Figure S2a.

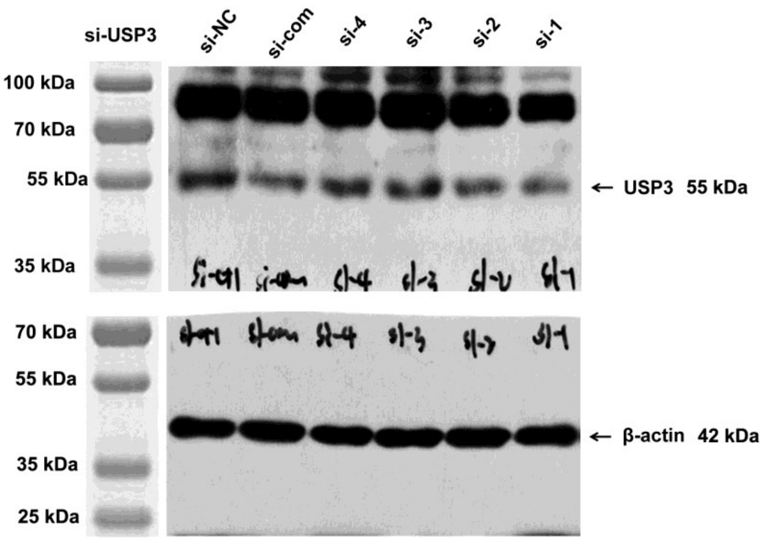

Supplementary Figure S18

Uncropped blots probed with USP3, SMAD4 and β-actin in Figure S2c.

Supplementary table S1. Primer list

Primers and sequences	
**Expressing vector**	
**p3.1-SMAD4 CDS clone**	
Forward	5’-TACAAGCTTATGGACAATATGTCTAT-3’
Reverse	5’-TAGCTCGAGTCAGTCTAAAGGTTGT-3’
**p3.1-USP3 CDS clone**	
Forward	5’-TGCAAGCTTATGGAGTGTCCACACCTG-3’
Reverse	5’-CGCCTCGAGTTAAAGTTTATCCGATC-3’
**Luciferase Reporter vector**	
**PGL3-SMAD4 3'UTR**	
Forward	5’-TCGTCTAGAGGTCTTTTACCGTTG-3’
Reverse	5’-GCGTCTAGAGTATGTTGAGATTCAG-3’
**PGL3-SMAD4 MUT1**	
Forward	5’-TTGGTATAATGTTTAAATCATGTATG-3’
Reverse	5’-ATCAAATCAAGTACAAAAATATCC-3’
**PGL3-SMAD4 MUT2**	
Forward	5’-TGTATAGAGAATTTAAGTAGAAAAG-3’
Reverse	5’-TGCCAATTGATATGATCATTGA-3’
**PGL3-USP3 3’UTR**	
Forward	5’-CGCTCTAGATACCTCCTCCAAATCATCATTC-3’
Reverse	5’-CGCTCTAGATTGTATTTGCAGGAC-3’
**PGL3-USP3 3’UTR MUT**	
Forward	5’-CTGAACATGGGCACCAACTAATTTTG-3’
Reverse	5’-TACCATTGACAAAACTAAATCCAAAATAGG-3’
**Real-time PCR primer**	
**GAPDH**	
Forward	5’-TGCACCACCACCTGCTTAGC-3’
Reverse	5’-GGCATGGACTGTGGTCATGAG-3’
**SMAD4**	
Forward	5’-GCTGCTGGAATTGGTGTTGATG-3’
Reverse	5’-AGGTGTTTCTTTGATGCTCTGTCT-3’
**USP3**	
Forward	5’-CAAGCTGGGACTGGTACAGAA-3’
Reverse	5’-GCAGTGGTGCTTCCATTTACTT-3’
**Dicer**	
Forward	5’-TGCTATGTCGCCTTGAATGTT-3’
Reverse	5’-AATTTCTCGATAGGGGTGGTCTA-3’

Supplementary table S2

Common microRNA response elements (MREs) predicted to be shared by USP3 and SMAD4 (http://www.microrna.org/)

Common miRNAs	No. of MREs in SMAD4	mirSVR scores of miRNA binds to SMAD4	No. of MREs in USP3	mirSVR scores of miRNA binds to USP3
**hsa-miR-224**	3	-0.5941	2	-1.5131
**hsa-miR-449a**	1	-0.5077	1	-0.2348
**hsa-miR-449b**	1	-0.5077	1	-0.2348
**hsa-miR-26b**	1	-0.1425	1	-1.2658
**hsa-miR-301a**	1	-0.2461	1	-0.4643
**hsa-miR-454**	1	-0.2439	1	-0.4609
**hsa-miR-130a**	1	-0.2332	1	-0.4543
**hsa-miR-130b**	1	-0.2332	1	-0.4227
**hsa-miR-301b**	1	-0.2295	1	-0.4643
**hsa-miR-146a**	1	-0.1722	1	-1.2170
**hsa-miR-146b-5p**	1	-0.1722	1	-1.2170
**hsa-miR-519d**	1	-0.1603	1	-1.3166
**hsa-miR-1297**	1	-0.1551	1	-1.2669
**hsa-miR-93**	1	-0.1587	1	-1.3155
**hsa-miR-106a**	1	-0.1557	1	-1.3149
**hsa-miR-106b**	1	-0.1557	1	-1.3105
**hsa-miR-17**	1	-0.1557	1	-1.3149
**hsa-miR-20a**	1	-0.1557	1	-1.3098
**hsa-miR-20b**	1	-0.1557	1	-1.3098
**hsa-miR-599**	1	-0.1395	1	-0.1237
**hsa-miR-208a**	1	-0.1234	1	-0.1901
**hsa-miR-208b**	1	-0.1210	1	-0.1901

Supplementary table S3

mirSVR scores of different MREs in SMAD4 and USP3 targeted by miR-224

Target mRNA by miR-224	MRE location	mirSVR score
**SMAD4**	1047-1053bp of SMAD4 3' UTR	-0.3769
**SMAD4**	1194-1200bp of SMAD4 3' UTR	-0.1054
**SMAD4**	5907-5913bp of SMAD4 3' UTR	-0.1118
**USP3**	134-140bp of USP3 3' UTR	-1.2221
**USP3**	266-271bp of USP3 3' UTR	-0.2910
